# Human Cysticercosis in Asia: A Systematic Review and Meta-Analysis

**DOI:** 10.18502/ijph.v49i10.4683

**Published:** 2020-10

**Authors:** Negar BIZHANI, Saeideh HASHEMI HAFSHEJANI, Neda MOHAMMADI, Mehdi REZAEI, Mohammad Bagher ROKNI

**Affiliations:** 1. Department of Medical Parasitology and Mycology, School of Public Health, Tehran University of Medical Sciences, Tehran, Iran; 2. Department of Epidemiology and Biostatistics, School of Public Health, Tehran University of Medical Sciences, Tehran, Iran; 3. Department of Forestry and Landscape Architecture, Konkuk University, Seoul, Republic of Korea

**Keywords:** Cysticercosis, *Taenia solium*, Human, Neglected tropical disease, Prevalence, Asia

## Abstract

**Background:**

Cysticercosis in among the neglected tropical disease caused by eating the egg of parasite *Taenia solium*. In this review, we aimed to verify the prevalence of human cysticercosis in different countries of Asia using systematic review and meta-analysis approach.

**Methods:**

Based of the protocol, reliable databases including PubMed, SCOPUS, Science Direct, Embase, and Cochrane Library from 1990–2018 were searched using a panel of keywords. Overall, 48 countries of Asia were searched in turn and data were analyzed using a category of statistical tests.

**Results:**

Out of 28 included studies, 586175 samples were collected and included in the data analysis. Based on the meta-analysis results, the overall pooled percent of cysticercosis was estimated 3.8% (95% CI: [2.0, 7.0]). According to the result of heterogeneity statistics including I-squared, chi-square, and tau-squared, it was statistically significant (Tau2 = 2.94, chi2 = 12733.31, *P*<0.001, I2 = 100%) therefore a random effect model was used to handle the heterogeneity of studies. To evaluate the trend of cysticercosis over the time, Cumulative meta-analysis was performed and the result showed that there was a minor upward tendency in the prevalence of cysticercosis over the time.

**Conclusion:**

Although, considering the religious culture and food habits in Asia, we might have expected to witness a low prevalence of human cysticercosis, but we noticed more or less significant infection in some countries of the region. Regarding the new feature of immigration and travel between countries, all authorities are advised to take measures on controlling and monitoring the disease.

## Introduction

One of diseases regarded as Neglected Tropical Diseases (NTD) by WHO is cysticercosis (1). Cysticercosis is a parasitic disease established after ingestion *Taenia solium* egg through contaminated food (2). Both humans and pigs might be infected with the disease. After ingestion the egg, oncospheres hatch in the small intestine, invade the intestinal wall and afterwards migrate to internal organs including striated muscles, brain, liver, and other tissues. There cysticerci is developed and results to cysticercosis (2). Many people in Asia consume pork, so it is possible that parasite’s eggs are spread everywhere, therefore cysticercosis might be occurred in Asia especially southeast Asia countries (3–6).

One of the most dangerous form of cysticercosis is Neurocysticercosis (NCC), which is occurred when the larvae of *T. solium* migrate to brain (2, 7, 8).

Considering that *T. solium* is transmitted via eating pork, and it is unusual in many Muslim countries in Asia, we expect to detect cysticercosis mostly in south-east countries including China (9, 10), Vietnam (11), India (12), Indonesia (4), Thailand (3) Korea (13), etc.

In this study, we aimed to collect and analyze data concerning the situation of human cysticercosis in Asia countries through searching authentic databases from 1990–2018. Animal cysticercosis and the prevalence of *T. solium* itself was not searched.

## Methods

### Search strategy

The databases including PubMed, SCOPUS, Science Direct, Embase, and Cochrane Library were searched using a panel of keywords, including, but not limited to, human cysticercosis, prevalence, epidemiology, and all Asia countries in turn (https://www.worldatlas.com/articles/how-many-countries-are-in-asia.html). The searched countries included Afghanistan, Armenia, Azerbaijan, Bahrain, Bangladesh, Bhutan, Brunei, Cambodia, China, Cyprus, East Timor, Georgia, India, Indonesia, Iran, Iraq, Israel, Japan, Jordan, Kazakhstan, Kuwait, Kyrgyzstan, Laos, Lebanon, Malaysia, Maldives, Mongolia, Myanmar, Nepal, North Korea, Oman, Pakistan, Philippines, Qatar, Saudi Arabia, Singapore, South Korea, Sri Lanka, State of Palestine, Syria, Tajikistan, Thailand, Turkey, Turkmenistan, United Arab Emirates, Uzbekistan, Vietnam and Yemen.

We included studies conducted between 1990–2018 published in English and relevant to the aim of the study. We screened at first the studies based on their title and abstract followed by availability of full text. For availability of abstracts only, we used them for Discussion section, if appropriated. Items such as books and Letter to the Editors were excluded from the study.

**Fig. 1:**
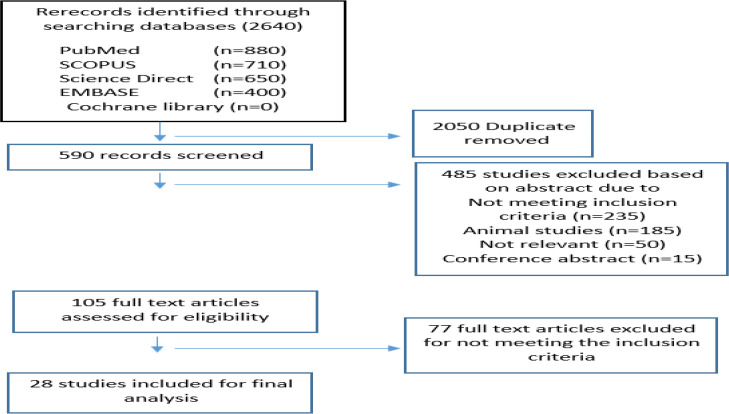
The flow diagram of search and selection of relevant articles

Inclusion criteria were studies 1) Conducted from 1990–2018; 2) Studies of English language 3) Availability of the full text; 4) Studies of Original or Review Article kind and 5) Human studies. Exclusion criteria were 1) Studies conducted regarding animals only; 2) Case Report studies. According to Prisma guideline (14), altogether, 2050 records were initially found through searching the databases mentioned already (Fig. 1).

After the initial screening, 77 articles were checked to verify the eligibility according to inclusion criteria, and eventually, 28 studies were included into the study.

### Statistical analysis

In the present study, the data were evaluated using R version 4.0.2 (R Foundation for Statistical Computing, Vienna, Austria.). The overall prevalence of cysticercosis was estimated using a point estimate and 95% confidence interval. The heterogeneity of studies were analysis using I-squared, Chi-square, and Tau-squared tests. The results with *P* value less than 0.05 were set statistically significant.

### Results and Discussion

**Fig. 2:**
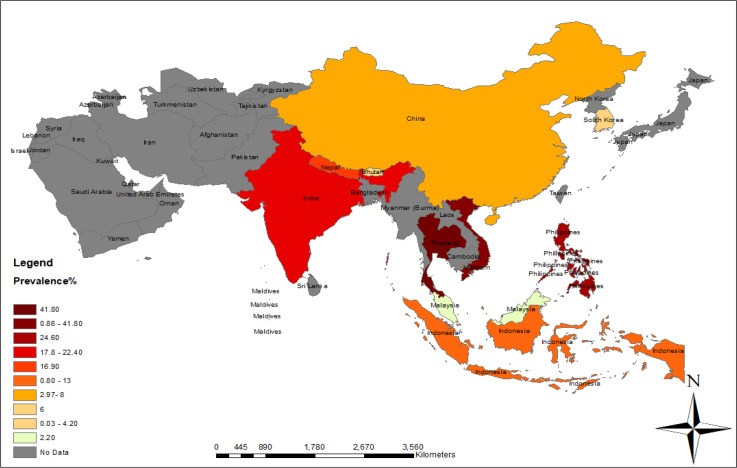
Distribution map of cysticercosis in Asia

As mentioned earlier, we noticed that cysticercosis is found mostly in Southeast Asia countries. The prevalence varied from 0.80% in Indonesia (15) to 41.8% in Thailand (3). The whole distribution of human cysticercosis is demonstrated in Fig. 2.

### Result of meta-analysis

**Fig. 3:**
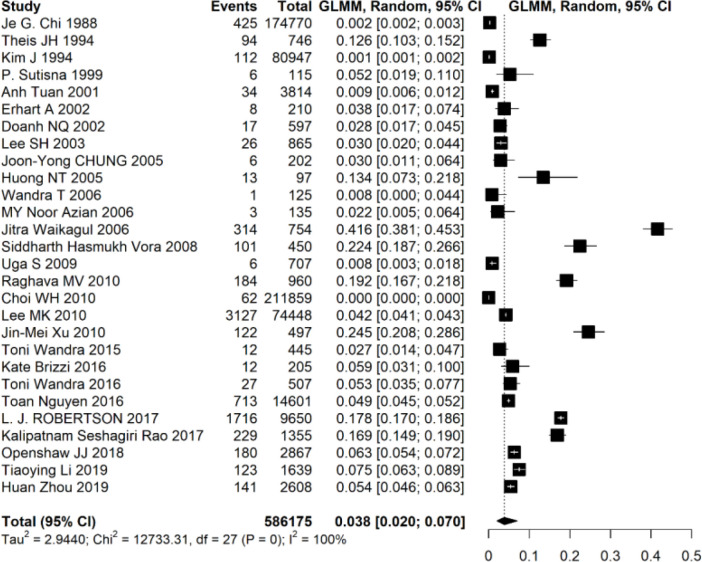
Forest plot showing the pooled prevalence of cysticercosis in Asia

Out of 28 included studies, 586175 samples were collected and included in the data analysis. Based on the meta-analysis results, the overall pooled percent of cysticercosis was estimated 3.8% (95% CI: [2.0, 7.0]). According to the result of heterogeneity statistics including I-squared, chi-square, and tau-squared, it was statistically significant (Tau2 = 2.94, chi2 = 12733.31, *P* value <0.001, I2 = 100%) therefore a random effect model was used to handle the heterogeneity of studies (Fig. 3).

**Fig. 4:**
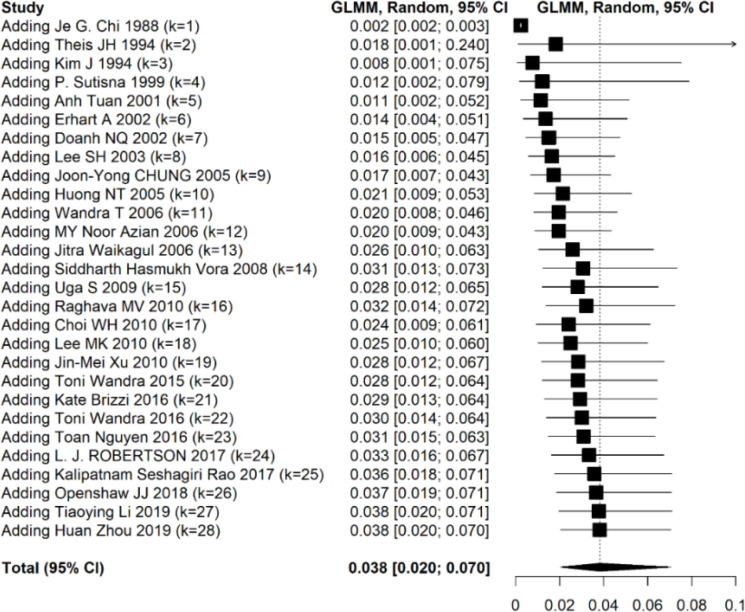
Cumulative forest plot showing the trend of cysticercosis prevalence in Asia

To evaluate the trend of cysticercosis over the time, Cumulative meta-analysis was performed and the result showed that there was a minor upward tendency in the prevalence of cysticercosis over the time (Fig. 4).

### China

NCC has been reported in China as affecting about 7 million people (16, 17). During a study on 2867 students in China, it was found that antibody to *T. solium* cysticercosis was present in 6% of subjects. In some schools, the prevalence was even 15% (16). Other studies showed human cysticercosis in China as 2.97% (10), 2.29% (18), and 4.32% (19). In minority areas of western Sichuan, China, from 2016 to 2017 a large study was conducted to detect the seroprevalence of cysticercosis in primary school-aged children. ELISA was used as serologic test. Eventually, form 1639 children examined, seropositivity was detected from 2.3% to 15.6% (overall 7.5%). It was found that children infected with *T. solium* showed more seropositivity than children without it (43.6% vs. 6.6%) (20). Zhou et al surveyed the rate of seropositivity for NCC in three mountainous areas in Sichuan Province of western China. From 2608 students, the seroprevalence of cysticercosis IgG antibodies was detected as 5.4%. The authors declared that sociodemographic factors, sources of pork and pig husbandry, as well as behavioral factors were contributed to establishing NCC (21).

### Vietnam

Cysticercosis has been reported from Vietnam as well. Although *T. solium* is endemic in this country but from 1994 to 2002 the prevalence of cysticercosis was less than 1% (11, 22). Of course, the reported prevalence was mostly based on animal cysticercosis. However, as for human cysticercosis a review article reported 13% prevalence (22). Altogether, about 250–400 patients infected with cysticercosis have been reported form Vietnam from 2006–2011 (23). Another study reported a prevalence of human cysticercosis of 5% (average 2.2%–7.2%) from 1999–2000 (24). Erhart et al reported human cysticercosis as 5.7% in a village in 1999, from them 9 cases had NCV (25). The only study in south of Vietnam reported 4.3% seropositivity among people for cysticercosis (26). These data show that Vietnam has problem with the disease and should monitor it. A formal report testifies that males were significantly more involved and the age group of 30-6 yr old had the highest rate of infection (23). Among risk factors mentioned for the disease in Vietnam, “the consumption of raw vegetables, drinking unboiled water, not washing hands before eating and outdoor defecation” may be considered (22).

### India

Although India has a low portion of consuming pork, but considering that the pigs are present in this country, we should expect infection with cysticercosis there. Especially that mostly poor people raise pigs so the sanitation is not observed properly (12). A prevalence of 4·5/1000 for NCC, has been reported in the peopled infected with epilepsy in Jammu and Kashmir, Northwest India (27). During a serological study in south India, 391 patients were examined from January 2011 to December 2015. Altogether 32.5% of cases were found seropositive for NCC, of which male cases with 59.1% seropositivity showed higher rate than females (40.9%) (28). Another study in south India, reported a seroprevalence of 1.28/1000 in urban areas and 1.02/1000 in rural areas for NCC (29). In all these studies, NCC has been attributed as a major cause of epilepsy. Considering the estimation of the health and economic burden of neurocysticercosis on people in India, a comprehensive study was conducted by Sing et al in India (30). The results showed that in terms of DALYs it was 1.73 per thousand people. An annual economic burden of 12.03 billion rupees was calculated as well. Vora et al reported 22.4% seroprevalence for 450 people from rural Goa, India examined for cysticercosis (31). The prevalence increased in parallel with age.

### Korea

Different seroprevalence rate has been reported for cysticercosis in Korea. A study by Lee et al Using ELISA test in 2001, detected 3.0% (26 cases) seropositivity for cysticercosis among 865 studied samples (32). Another long period study conducted in Korea from 1993 to 2006, examined 74448 samples using ELISA test. It was showed that seropositivity was decreased from 8.3% (1993) to 2.2% (2006) for cysticercosis (33). Age group of 50–59 years encompassed the highest positivity as 21.2%, besides to people coming from the Seoul (Capital) as 55.9%. It shows that tissue invading parasitic infections is an important issue in Korea and should be regarded as significant phenomenon. Cho et al analyzed 211859 biopsy specimens in Seoul, Korea during 1984–2005 and found 62 cases of cysticercosis (34), out of them, 55 cases were detected in sub-cutaneous tissues or the central nerve system. They reported that from the year 2000, no case of cysticercosis was found and all reported cases belonged to 1984–1988. During another long period study from 1980–89 with 80947 biopsy specimens conducted in Korea, 112 cases of cysticercosis were reported (35).

### Indonesia

Altogether, from 1985 until 2006, the seroprevalence on cysticercosis has been reported as 5.2%–21% in this country (15, 36–38). From the early 1990s, *T. solium* was presented in Indonesia, therefore, cases of NCC were reported there more or less. However a joint project supported financially by Japan, regarding the control of NCC initiated from 1996 (39). Among the regions famous for NCC is Papua (formerly Irian Jaya), because of having some cases of outbreaks due to NCC (39). Although a 10-year project to control the disease has been started by the government from 1990, yet it is labeled as high endemic are for NCC and subcutaneous cysticercosis (36, 40). Besides, NCC was common in Bali from 1990s (39, 41). After taking health measurement by the government, the cases of NCC have been reduced significantly and considered as sporadic. The issue of immigrants infected with NCC or T. solium cause spreading the disease to different parts of Indonesia (39). Feeding from infected pigs, looking for job by many unemployed infected individuals especially in dry session have been mentioned as factors assisting to spread NCC (39). Moreover, a local food entitled *lawar*, prepared with uncooked beef or pork is reported as another factor in the region. Bali is a favorite place for tourists so there is the risk of infection with the disease. Theis et al conducted a study on 746 residents in Bali to find the anti-Cysticercus antibodies, from which, 94 cases (13%) showed seropositivity (37). They concluded that epilepsy might be an output of NCC in the area. Another study from rural areas of Bali showed seroprevalence of human cysticercosis by immunoblot as 1.65% (6/363) (38). Some undocumented cases of subcutaneous cysticercosis have been reported from the local clinics as well. Other reports showed that 45.8% of people, 70.4% of pigs, and 10.9% of local dogs were seropositive for cysticercosis in Papua (36, 42, 43). These figure shows that in terms of cysticercosis the situation in Indonesia and especially Papua needs more attention by local authorizes and international involved organizations.

### Other countries

Considering that cysticercosis in transmitted via eating parasite’s egg released by pig, we expect that the disease is present in only some limited countries depending on the religious and food habitats and cultures. Anyway, other countries in Asia, more or less show a degree of infection with cysticercosis.

In Bhutan, people with epilepsy were investigated for NCC by ELISA and EITB. Results showed that 6% were definitely infected with NCC (44). Twenty percent were reported as probable NCC. Rao et al conducted a study in Nepal from 2033–2015 on children. They reported 229 (16.90%) case of NCC among the 1355 cases of seizure disorders (8). The authors have expressed their satisfaction of the trend of NCC during the last years in Nepal. In Thailand we have report from Waikagul et al (3). Accordingly cysticercosis was reported as 41.8% via immunoblot technique from 2000–2005. They believe that the rate of infection already reported in literature is underestimate and needs more verification. In Thailand people are used to consume raw/half-cooked meat dishes so the risk of taeniasis and cysticercosis would be considered by authorities. Noor Azian et al verified the situation of cysticercosis in Malaysia among a rural village. They reported a seropositivity as 2.2% among 135 studied cases (6). In the Philippine, we have the situation of endemic for cysticercosis (45). In a study conducted in rural areas, the seroprevalence of 24.6% was detect among 497 studies subjects (45). This shows more necessity for considering the disease in this country.

## Conclusion

Indeed we might have expected a low prevalence of human cysticercosis in the region in comparison with other regions, but the rate of infection shows considerable range. It varied from 0.8% to 41.8%. Of course, countries where pork can be seen in the matrix of people lives, showed more infection. Increasing rate of travel and immigration between countries, looking for job and better job in countries where cysticercosis had not been reported already, changing food habits because of shortage of food materials, increasing political turmoils in most countries of the region which force people to move to other countries, and some similar reasons have caused to spread the NTDs, among them human cysticercosis and NCC are considered very important. It is due to pathological effects this disease brings for patients.

## Ethical considerations

Ethical issues (Including plagiarism, informed consent, misconduct, data fabrication and/or falsification, double publication and/or submission, redundancy, etc.) have been completely observed by the authors.

## References

[1] World Health Organization (2020). Neglected tropical diseases. www.who.int/neglected_diseases/diseases/en/

[2] Ndimubanzi P Carabin H Budke C (2010). A Systematic Review of the Frequency of Neurocyticercosis with a Focus on People with Epilepsy. PLoS Negl Trop Dis, 4:e870. 21072231 10.1371/journal.pntd.0000870 PMC2970544

[3] Waikagul J Dekumyoy P Anantaphruti MT (2006). Taeniasis, cysticercosis and echinococcosis in Thailand. Parasitol Int, 55 Suppl:S175–80. 16338166 10.1016/j.parint.2005.11.027

[4] Wandra T Ito A Swastika K Dharmawan NS Sako Y Okamoto M (2013). Taeniases and cysticercosis in Indonesia: past and present situations. Parasitology, 140:1608–16. 23965293 10.1017/S0031182013000863

[5] Yamasaki H (2013). Current status and perspectives of cysticercosis and taeniasis in Japan. Korean J Parasitol, 51:19–29. 23467264 10.3347/kjp.2013.51.1.19 PMC3587745

[6] Noor Azian MY Hakim SL Sumiati A Norhafizah M (2006). Seroprevalence of cysticercosis in a rural village of Ranau, Sabah, Malaysia. Southeast Asian J Trop Med Public Health, 37:58–61. 16771213

[7] Piryani RM Kohli SC Shrestha G Shukla A Malla TB (2007). Human neurocysticercosis managed at Nepalganj Medical College, Teaching Hospital, Kohalpur, Nepal. Kathmandu Univ Med J (KUMJ), 5:518–20. 18604086

[8] Rao KS Adhikari S Gauchan E (2017). Time trend of neurocysticercosis in children with seizures in a tertiary hospital of western Nepal. PLoS Negl Trop Dis, 11:e0005605. 28489921 10.1371/journal.pntd.0005605 PMC5440051

[9] Li T Craig PS Ito A (2006). Taeniasis/cysticercosis in a Tibetan population in Sichuan Province, China. Acta Trop, 100:223–31. 17166477 10.1016/j.actatropica.2006.11.003

[10] Chung JY Eom KS Yang Y (2005). A seroepidemiological survey of *Taenia solium* cysticercosis in Nabo, Guangxi Zhuang Autonomous Region, China. Korean J Parasitol, 43:135–9. 16340302 10.3347/kjp.2005.43.4.135 PMC2712017

[11] Ng-Nguyen D Noh J Breen K Stevenson MA Handali S Traub RJ (2018). The epidemiology of porcine Taenia solium cysticercosis in communities of the Central Highlands in Vietnam. Parasit Vectors, 11:360. 29929529 10.1186/s13071-018-2945-y PMC6014001

[12] Robertson LJ Joshi H Utaaker KS Kumar A Chaudhary S Goyal K Sehgal R (2017). Changes in the seroprevalence of cysticercosis in suspected patients in Chandigarh, India between 1998 and 2014: analysis of 17 years of data. Epidemiol Infect, 145:1159–1167. 28091347 10.1017/S0950268816003356 PMC9507844

[13] Chai JY (2013). Human taeniasis in the Republic of Korea: hidden or gone? Korean J Parasitol, 51:9–17. 23467688 10.3347/kjp.2013.51.1.9 PMC3587755

[14] Moher D Liberati A Tetzlaff J Altman D (2009). Preferred Reporting Items for Systematic Reviews and Meta-Analyses: the PRISMA statement. Br Med J, 8:336–341. PMC3090117 21603045

[15] Wandra T Depary AA Sutisna P (2006). Taeniasis and cysticercosis in Bali and North Sumatra, Indonesia. Parasitol Int, 55 Suppl:S155–60. 16376140 10.1016/j.parint.2005.11.024

[16] Openshaw JJ Medina A Felt SA Li T Huan Z Rozelle S Luby SP (2018). Prevalence and risk factors for Taenia solium cysticercosis in school-aged children: A school based study in western Sichuan, People’s Republic of China. PLoS Negl Trop Dis, 12:e0006465. 29738570 10.1371/journal.pntd.0006465 PMC5959190

[17] Coyle CM Mahanty S Zunt JR (2012). Neurocysticercosis: neglected but not forgotten. PLoS Negl Trop Dis, 6:e1500. 22666505 10.1371/journal.pntd.0001500 PMC3362619

[18] Li Y Wu F F. Z (1996). The first survey on cysticercosis in humans in Nangang Region of Heliongjiang Province, China. Dis Monitor, 11.

[19] Ji K Tong S Liu Y (1996). Serological epidemiological surveys on cysticercosis in humans in Haerbin Region of Helongjiang Province, China. Chin J Parasit Dis Control, 9.

[20] Li T Chen X Wang H Openshaw JJ Zhong B Felt SA Ito A Luby SP (2019). High prevalence of taeniasis and Taenia solium cysticercosis in children in western Sichuan, China. Acta Trop, 199:105133. 31415736 10.1016/j.actatropica.2019.105133

[21] Zhou H Wang Q Zhou J Li T Medina A Felt SA Rozelle S Openshaw JJ (2019). Structural Equation Modeling (SEM) of Cysticercosis in School-Aged Children in Tibetan Rural Farming Areas of Western China: Implications for Intervention Planning. Int J Environ Res Public Health, 16:780. 10.3390/ijerph16050780 PMC6427563 30836642

[22] Ng-Nguyen D Stevenson MA Traub RJ (2017). A systematic review of taeniasis, cysticercosis and trichinellosis in Vietnam. Parasit Vectors, 10:150. 28320455 10.1186/s13071-017-2085-9 PMC5359969

[23] Vietnam MoHo (2012). Taeniasis and Cysticercosis. Rev. Work. helminthiasis Control Act. period 2006–2011 Implement. Work. period 2012–2015.

[24] Doanh NQ Kim NT De NV NN. L (2002). Result of survey on taeniasis and cysticercosis humans and pigs in Bac Ninh and Bac Kan provinces. Vet Sci Tech, 9:46–9.

[25] Erhart A Dorny P Văn Đệ N (2002). Taenia solium cysticercosis in a village in northern Viet Nam: Seroprevalence study using an ELISA for detecting circulating antigen. Trans R Soc Trop Med Hyg, 96:270–2. 12174775 10.1016/s0035-9203(02)90095-7

[26] Anh Tuan P Dung TTK Nhi VA (2001). Seroepidemiological investigation of cysticercosis in the southern provinces. J Malar Parasite Dis Control, 4:81–7.

[27] Raina SK Razdan S Pandita K Sharma R Gupta V Razdan S (2012). Active epilepsy as indicator of neurocysticercosis in rural northwest India. Epilepsy Res Treat, 2012: 802747. 22957243 10.1155/2012/802747 PMC3420514

[28] Thamilselvan P Muthuraman KR Mandal J Parija SC (2016). Rising trends of neurocysticercosis: A serological report from tertiary-care hospital in South India. Trop Parasitol, 6:141–146. 27722103 10.4103/2229-5070.190832 PMC5048701

[29] Rajshekhar V Raghava MV Prabhakaran V Oommen A Muliyil J (2006). Active epilepsy as an index of burden of neurocysticercosis in Vellore district, India. Neurology, 67:2135–9. 17190933 10.1212/01.wnl.0000249113.11824.64

[30] Singh BB Khatkar MS Gill JPS Dhand NK (2017). Estimation of the health and economic burden of neurocysticercosis in India. Acta Trop, 165:161–169. 26802489 10.1016/j.actatropica.2016.01.017

[31] Vora SH Motghare DD Ferreira AM Kulkarni MS Vaz FS (2008). Prevalence of human cysticercosis and taeniasis in rural Goa, India. J Commun Dis, 40:147–50. 19301700

[32] Lee S Kim M Back B Choi J Kim T Hwang Y (2003). Analysis of Parasite-Specific-Antibody Positive Patients for Clonorchis sinensis, Paragonimus westermani, Cysticercus and Sparganum using ELISA. Ann Lab Med, 23:126–131.

[33] Lee MK Hong S-J Kim HR (2010). Seroprevalence of tissue invading parasitic infections diagnosed by ELISA in Korea. J Korean Med Sci, 25:1272–1276. 20808668 10.3346/jkms.2010.25.9.1272 PMC2923801

[34] Choi WH Chu JP Jiang M (2010). Analysis of parasitic diseases diagnosed by tissue biopsy specimens at KyungHee Medical Center (1984–2005) in Seoul, Korea. Korean J Parasitol, 48:85–8. 20333293 10.3347/kjp.2010.48.1.85 PMC2843854

[35] Kim J Chung WS Cho KH (1994). [Status of parasitic infection diagnosed by surgical biopsy in Kwangju and Chollanam-do]. Korean J Parasitol, 32:93–100. 8025038 10.3347/kjp.1994.32.2.93

[36] Wandra T Ito A Yamasaki H Suroso T Margono SS (2003). *Taenia solium* cysticercosis, Irian Jaya, Indonesia. Emerg Infect Dis, 9:884–5. 12899138 10.3201/eid0907.020709 PMC3023452

[37] Theis JH Goldsmith RS Flisser A (1994). Detection by immunoblot assay of antibodies to *Taenia solium* cysticerci in sera from residents of rural communities and from epileptic patients in Bali, Indonesia. Southeast Asian J Trop Med Public Health, 25:464–8. 7777908

[38] Sutisna IP Fraser A Kapti IN (1999). Community prevalence study of taeniasis and cysticerosis in Bail, Indonesia. Trop Med Int Health, 4:288–94. 10357865 10.1046/j.1365-3156.1999.00394.x

[39] Wandra T Swastika K Dharmawan NS (2015). The present situation and towards the prevention and control of neurocysticercosis on the tropical island, Bali, Indonesia. Parasit Vectors, 8:148. 25881045 10.1186/s13071-015-0755-z PMC4356148

[40] Wandra T Sudewi AA Swastika IK (2011). Taeniasis/cysticercosis in Bali, Indonesia. Southeast Asian J Trop Med Public Health, 42:793–802. 22299461

[41] Ito A Wandra T Yamasaki H (2004). Cysticercosis/taeniasis in Asia and the Pacific. Vector Borne Zoonotic Dis, 4:95–107. 15228810 10.1089/1530366041210756

[42] Wandra T Subahar R Simanjuntak GM (2000). Resurgence of cases of epileptic seizures and burns associated with cysticercosis in Assologaima, Jayawijaya, Irian Jaya, Indonesia, 1991–95. Trans R Soc Trop Med Hyg, 94:46–50. 10748897 10.1016/s0035-9203(00)90433-4

[43] Wandra T Sutisna P Dharmawan NS Margono SS Sudewi R Suroso T Craig PS Ito A (2006). High prevalence of *Taenia saginata* taeniasis and status of Taenia solium cysticercosis in Bali, Indonesia, 2002–2004. Trans R Soc Trop Med Hyg, 100:346–53. 16199069 10.1016/j.trstmh.2005.06.031

[44] Brizzi K Pelden S Tshokey T (2016). Neurocysticercosis in Bhutan: a cross-sectional study in people with epilepsy. Trans R Soc Trop Med Hyg, 110:517–526. 27794094 10.1093/trstmh/trw066 PMC5444516

[45] Xu J-M Acosta LP Hou M (2010). Seroprevalence of Cysticercosis in Children and Young Adults Living in a Helminth Endemic Community in Leyte, the Philippines. J Trop Med, 2010:603174. 20368794 10.1155/2010/603174 PMC2846682

